# Template Effect of the Graphene Moiré Lattice on Phthalocyanine Assembly

**DOI:** 10.3390/molecules22050731

**Published:** 2017-05-03

**Authors:** Nicolas Néel, Jörg Kröger

**Affiliations:** Institut für Physik, Technische Universität Ilmenau, D-98693 Ilmenau, Germany; joerg.kroeger@tu-ilmenau.de

**Keywords:** graphene, phthalocyanine, template, scanning tunnelling microscopy

## Abstract

Superstructures of metal-free phthalocyanine (2H-Pc) molecules on graphene-covered Ir(111) have been explored by scanning tunnelling microscopy. Depending on the sub-monolayer coverage different molecular assemblies form at the surface. They reflect the transition from a graphene template effect on the 2H-Pc arrangement to molecular superstructures that are mainly governed by the intermolecular coupling.

## 1. Introduction

The impetus to template-guided growth is the long-range replication of building blocks in a controllable and reproducible manner. Data storage at the ultimate size [1,2], energy conversion [3] as well as heterogeneous catalysis [4,5,6,7] represent relevant technological fields that may benefit from such growth methods.

Different approaches to steering the deposition and adsorption of material on surfaces have been suggested, such as the use of strained layers and dislocations [8,9], vicinal surfaces [10,11,12,13,14,15,16,17,18] and supramolecular architectures [19,20,21]. Other templates exploit the spatial variation of the electronic structure [22,23] and moiré lattices [24,25] to guide the adsorption.

The template effect of moiré superstructures plays a particularly important role for two-dimensional materials, e.g., graphene and hexagonal boron nitride on various surfaces. Indeed, adsorbed graphene [26,27,28,29] and hexagonal boron nitride [30] form moiré lattices and represent appropriate templates for guiding the adsorption and intercalation of metal clusters [31,32,33,34,35,36,37,38,39,40,41,42] as well as of molecules [43,44,45,46,47,48,49,50,51,52,53]. A variety of superstructures, e.g., honeycomb and kagome networks, have been reported, which are intimately related to the different chemical activities of the adsorption sites [54]. For instance, the moiré pattern of graphene on various metal surfaces reflects the residing of graphene C${}_{6}$ rings at face-centered cubic (fcc), hexagonal close-packed (hcp) and on-top metal lattice sites. The distance of graphene to fcc, hcp and on-top sites depends on the graphene–metal interaction, which in turn determines the graphene corrugation on the specific metal surface. Both the local graphene–metal hybridization and the geometric corrugation of the moiré lattice are important for the adsorption energy.

Metal phthalocyanine (M-Pc) molecules have often been used to probe the adsorption energy landscape on graphene. These molecules consist of a central metal ion surrounded by an organic macrocycle of four pyrrole and four benzene groups. Planar arrays of M-Pc molecules on graphene with different structural motifs have been reported. The emerging picture that may be inferred from these adsorption studies is the formation of kagome superstructures when the graphene–metal hybridzation is high, as observed from Fe-Pc, Mn-Pc, Ni-Pc, 2H-Pc on graphene-covered Ru(0001) [43,55]. In contrast, densely packed two-dimensional islands with nearly square unit cells form on graphene with low coupling to the metal substrate, as observed from Fe-Pc molecules on graphene-covered Pt(111) [55] and Co-Pc on graphene-covered Ir(111) [56].

In this article we present our findings on coverage-dependent 2H-Pc assemblies on graphene-covered Ir(111). At low sub-monolayer coverage clusters of 2H-Pc dimers and trimers are observed whose constituents favour the adsorption on fcc and hcp regions of the graphene lattice. Higher sub-monolayer coverages reflect the continuation of preferably occupying fcc and hcp regions by forming a honeycomb array of 2H-Pc. Close to completion of the molecular monolayer a densely packed molecular superstructure with a nearly square unit cell is observed. These results contrast the aformentioned emerging picture of compact M-Pc and 2H-Pc arrays on graphene with low moiré-induced corrugation. We suggest that this deviation arises from the mismatch of the 2H-Pc dimension and the moiré periodicity on Ir(111). Moreover, our findings unveil the transition from the graphene template effect at low sub-monolayer coverage to a regime at higher coverage where the molecule–molecule coupling dominates over the molecule–graphene interaction.

## 2. Results and Discussion

**Figure 1:**
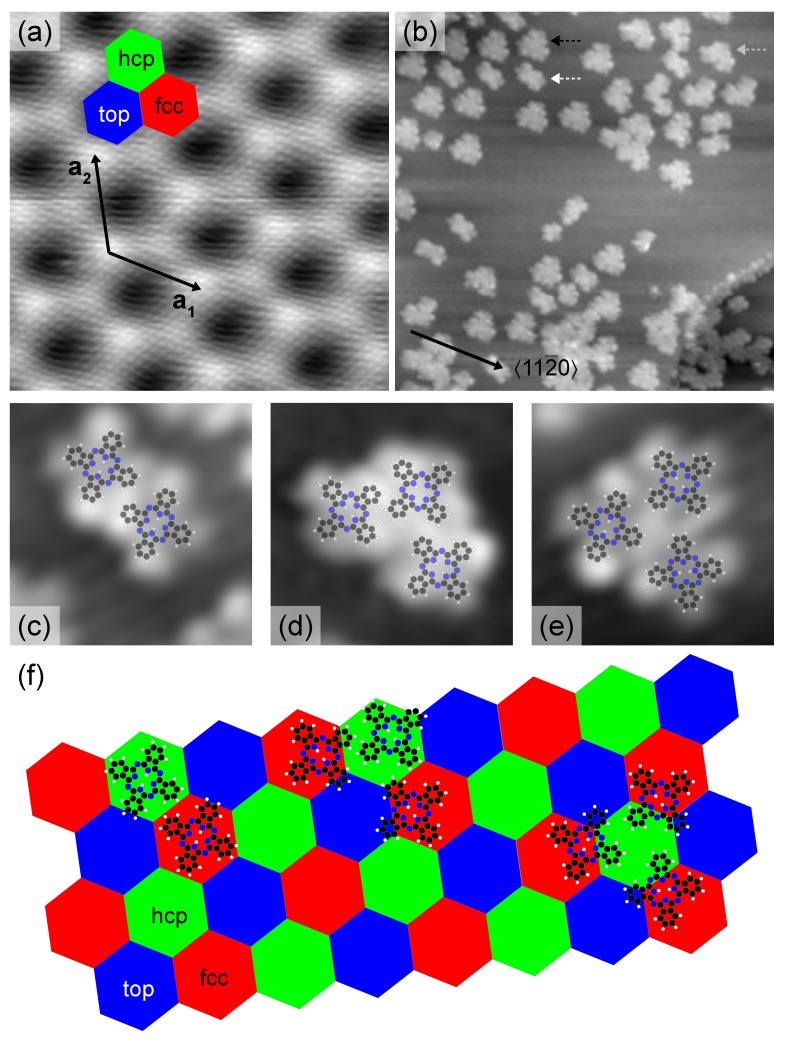
(**a**) STM image of graphene-covered Ir(111) (3nA, 0.175V, $10\times 10\;{\mathrm{nm}}^{2}$). The graphene lattice is visible as a hexagonal array of protrusions with lattice constant ≈0.25nm. The moiré superlattice is spanned by $a_{1}$, $a_{2}$ with $|a_{1}\left|=\right|a_{2}|\approx 2.5\;\mathrm{nm}$. The moiré lattice vectors $a_{1}$, $a_{2}$ are aligned with graphene $\langle 11\bar{2}0\rangle$ crystallographic directions. Red, green and blue hexagons indicate fcc, hcp and on-top regions of the graphene lattice, respectively; (**b**) STM image of graphene-coverd Ir(111) after deposition of 0.3ML of 2H-Pc (5pA, 1V, $37\times 37\;{\mathrm{nm}}^{2}$); Dashed arrows indicate 2H-Pc dimers (white) and trimers, which adopt the shape of isosceles (grey) and equilateral (black) triangles. (**c**–**e**) Close-up STM images (5pA, 1V, $4.4\times 4.4\;{\mathrm{nm}}^{2}$) of a 2H-Pc dimer (**c**), 2H-Pc trimer with isosceles (**d**) and equilateral (**e**) triangular shape. A stick-and-ball sketch of the molecule illustrates the molecular orientation; (**f**) Sketch of the suggested adsorption geometry of 2H-Pc dimers and trimers on graphene-covered Ir(111). The hexagonal tiling indicates the moiré pattern with fcc (red), hcp (green), on-top (blue) stacking regions.

Figure 1a shows a scanning tunnelling microscope (STM) image of graphene on Ir(111). Two periodic patterns are observed. The hexagonal arrangement of circular protrusions with a periodicity of ≈0.25 nm arises due to the graphene atomic lattice. The larger periodicity visible in the STM image is ≈2.5 nm and reflects the graphene moiré lattice on Ir(111). The moiré superstructure is due to the different lattice constants of graphene, 0.2452nm, and Ir(111), 0.2715nm [57]. The observed alignment of the atomic and moiré lattices together with the moiré periodicity indicate that $\langle 11\bar{2}0\rangle$ graphene crystallographic directions coincide with $\langle 1\bar{1}0\rangle$ Ir(111) crystallographic directions. The different apparent heights of the moiré lattice are due to C${}_{6}$ rings of graphene residing at or close to specific Ir(111) sites. At low bias voltage fcc and hcp (on-top) sites appear bright (dark) in STM images [58]. The brightest contrast reflects C${}_{6}$ rings encircling fcc Ir(111) lattice sites (red hexagon in Figure 1a), while the protrusion with less contrast is due to graphene regions where C${}_{6}$ rings encircle hcp Ir(111) lattice sites (green hexagon in Figure 1a). The depressions are assigned to C${}_{6}$ rings enclosing an on-top Ir(111) site (blue hexagon in Figure 1a).

Deposition of 2H-Pc with a coverage of ≈30% of a monolayer (ML) leads to STM images as presented in Figure 1b. 1mL corresponds to the surface density of 2H-Pc for the close-packed assembly at high coverage, $0.51\;{\mathrm{nm}}^{-2}$. A rather low corrugation of the adsorption energy of the molecules to graphene was evidenced by the high mobility of the molecules during scanning. Stable imaging was only achieved for large tip–surface distances, that is, for bias voltages (tunneling currents) on the order of 1V (1pm). Moreover, a low binding is in agreement with previous reports [59,60]. The molecules form dimers and trimers while large regions of graphene remain unoccupied. Monomers, i.e., single 2H-Pc molecules, have not been observed, which indicates an attractive interaction between closely separated molecules (*vide infra*). The arrangement of individual 2H-Pc in dimers and trimers depends on the cluster size and shape. In dimers (Figure 1c) 2H-Pc molecules exhibit similar orientation. Defining a molecular axis by two opposite isoindole groups we find the alignment of one of the molecular axis with $\langle 11\bar{2}0\rangle$. The intermolecular distance as inferred from the separation of the molecule centers is ≈1.4nm, which corresponds to the molecule–molecule distance in the close-packed superstructure at high coverage (*vide infra*). Trimers of 2H-Pc adopt two different configurations (Figure 1d,e). They occur as isosceles (Figure 1d) and equilateral (Figure 1e) triangles. Linear chains of three (or more) 2H-Pc have not been observed. In trimers with isosceles triangular shape at least two molecules show the same orientation, while in trimers with equilateral triangular shape the individual molecules exhibit different orientations. In all trimers one molecular axis of each constituent is aligned with $\langle 11\bar{2}0\rangle$. The intermolecular distance is ≈1.5 nm for all trimers.

The 2H-Pc dimers and trimers tend to locally arrange in a nearly hexagonal lattice with nearest-neighbour (next-nearest neighbour) distances of ≈2.5 nm (≈4.3 nm). This assembly corresponds well to the arrangement of fcc, hcp and on-top graphene regions (Figure 1a). We therefore suggest that at this coverage 2H-Pc molecules preferentially adsorb at specific sites of the graphene moiré lattice (Figure 1f). On-top regions may be excluded as adsorption sites since calculations unveiled that these regions exhibit poor chemical reactivity due to the virtual absence of graphene—metal charge transfer [58,61,62]. The similar charge transfer for fcc and hcp regions hampers the clear-cut assignment of preferential adsorption to these regions. To this end additional contributions to the adsorption energy, such as intermolecular interactions and electric dipoles, would have to be considered.

In 2H-Pc dimers (Figure 1c) the molecules occupy adjacent fcc and hcp graphene regions since the intermolecular separation is in agreement with the distance between fcc and hcp regions, 1.48nm. Weak intermolecular C–H …N bonds [63] most likely couple the two 2H-Pc molecules. Long-range interactions mediated by electronic surface states as observed on the (111) surfaces of noble metals [63] are unlikely since a modulation of the density of states due to surface electron interference was not discernible in STM images.

By analyzing the orientation of molecular trimers that adopt an isosceles triangular shape (Figure 1d) the preferred stacking region for adsorption may be identified. While the long side of the isosceles triangles is aligned with $\langle 11\bar{2}0\rangle$, the short sides enclose an angle of $30^{\circ }$ with it. Moreover, the orientation of these trimers is uniform, that is, isosceles triangles rotated in the surface plane by integer multiples of $60^{\circ }$ with respect to the orientation of trimers visible in Figure 1 were not observed. These results are only compatible with the two molecules that form the long side of the trimer residing at fcc regions while the remaining one occupies the adjacent hcp region (Figure 1f).

The edges of 2H-Pc trimers with an equilateral triangular shape are oriented parallel to crystallographic directions (Figure 1e). Individual 2H-Pc molecules belonging to such trimers could therefore occupy fcc or hcp graphene regions. The results obtained for isosceles triangular trimers evidence that the fcc site is preferred. Therefore, it is likely that 2H-Pc molecules of equilateral triangular trimers occupy fcc sites. Occupation of hcp sites would moreover imply that the on-top region is covered by significant portions of the molecules, which, however, is not favoured.

The presence of dimers and trimers of 2H-Pc on graphene-covered Ir(111) differs from the low-coverage 2H-Pc assembly reported from graphene-covered Ru(0001) [55]. On the latter surface small patches of a kagome lattice were observed. As will be discussed below the smaller moiré lattice constant of graphene-covered Ir(111) does not allow the formation of a kagome lattice of 2H-Pc on this surface.

Before presenting the findings for higher coverage a comment on the cluster distribution appears to be noteworthy. The top left part of Figure 1b shows that 2H-Pc clusters (locally) occupy next-nearest moiré lattice sites. A coalescence of clusters by additionally adsorbed molecules may be inhibited by a repulsive interaction. Most likely, H–H steric repulsion between the bridging 2H-Pc and one of the clusters is at the origin of this interaction. If the two bridged molecules of the clusters exhibit different orientations one of the phenyl groups of the bridging 2H-Pc points towards a phenyl group of one of the 2H-Pc belonging to a cluster, which favours steric repulsion. However, cluster coalescence may occur if the orientation of bridged cluster molecules are similar, which enables attractive C–H …N bonds to the bridging 2H-Pc (lower part of Figure 1b).

**Figure 2:**
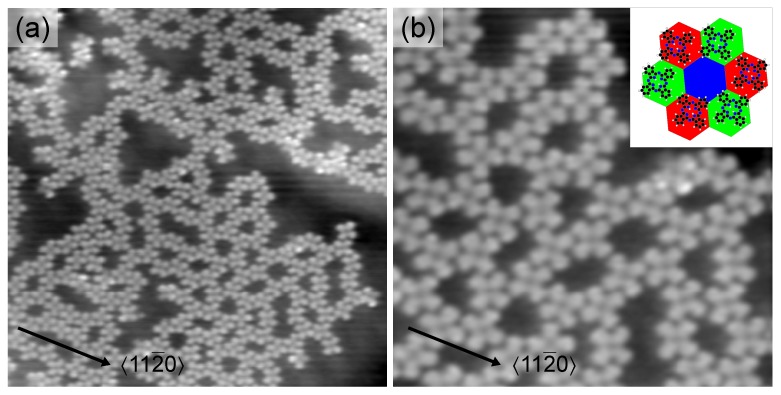
(**a**) STM image of graphene-covered Ir(111) after deposition of 0.6ML of 2H-Pc (10pA, 1V, $30\times 30\;{\mathrm{nm}}^{2}$). Locally the molecules form a honeycomb lattice; (**b**) Close-up STM image of the molecular honeycomb superstructure. Inset: Sketch of the suggested adsorption configuration in the honeycomb lattice. Fcc (red hexagon) and hcp (green) graphene regions are occupied by 2H-Pc. On-top sites (blue) remain unoccupied and are the pores of the honeycomb superlattice.

Increasing the molecular coverage leads to the occupation of fcc as well as of hcp graphene sites while on-top regions remain unoccupied (Figure 2a). The resulting honeycomb arrangement of 2H-Pc molecules reflects the graphene moiré pattern. The close-up view of the molecular honeycomb lattice presented in Figure 2b shows that a ring of six 2H-Pc molecules encloses a central unoccupied region. On the basis of the low-coverage results we suggest that the unoccupied region is the graphene on-top site. Alternately, fcc and hcp graphene regions are occupied by the 2H-Pc molecules forming the rings of the honeycomb lattice (inset to Figure 2b). Within a specific honeycomb ring the individual molecules adopt similar orientations. This orientation may change from ring to ring. However, all orientations are characterized by one molecular axis coinciding with a graphene crystallographic direction. The distance of adjacent ring molecules is nearly the same as observed from the close-packed superstructure at high coverage.

Before discussing the high-coverage results a comment on the observed honeycomb superstructure appears to be appropriate. Deviating from our findings 2H-Pc on graphene-covered Ru(0001) forms a kagome rather than a honeycomb lattice [43]. The kagome superstructure represents a trihexagonal tiling of the surface where 2H-Pc molecules reside at the corners of equilateral triangles and regular hexagons. In order to form a molecular kagome lattice on graphene two stacking regions of the moiré pattern—fcc and hcp—should serve as preferred adsorption sites, which for graphene-covered Ru(0001) is fulfilled as well as for Ir(111). Additionally, the molecule dimensions must match the moiré lattice constant. The moiré periodicity of graphene on Ru(0001) is ≈3 nm. Therefore, the distance between adjacent molecules of the kagome superstructure will not go below the mutual separation observed from the close-packed molecular array. In contrast, the moiré periodicity of graphene on Ir(111) is ≈2.5 nm, which would force adjacent 2H-Pc molecules in a kagome lattice to be separated by less than their distance in the close-packed arrangement. Such an arrangement is not favored due to repulsive H–H interactions between benzene groups of neighbouring molecules.

**Figure 3:**
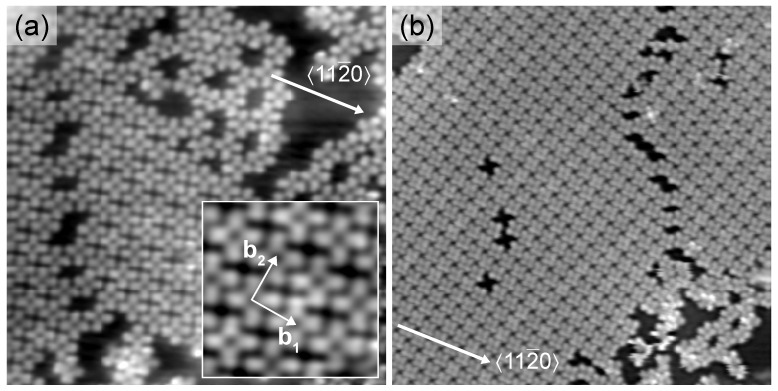
(**a**) STM image of graphene-covered Ir(111) after deposition of 0.75ML of 2H-Pc (10pA, 1V, $21\times 21\;{\mathrm{nm}}^{2}$). Inset: Close-up view of (**a**) with $b_{1}$, $b_{2}$ indicating the unit cell vectors of the densely packed superstructure; (**b**) STM image of a large molecular island exhibiting the close-packed 2H-Pc arrangement (10pA, 1V, $42\times 34\;{\mathrm{nm}}^{2}$).

One could expect that the further increase of the coverage may give rise to the occupation of the vacant on-top regions of graphene, which would then result in a hexagonal 2H-Pc superstructure reflecting the moiré pattern. Filling the pores of a honeycomb molecular superlattice has indeed been reported before [52]. Here, however, adding further molecules leads to a molecular superstructure with different symmetry (Figure 3). 2H-Pc molecules are arranged in an essentially square lattice (inset to Figure 3a) with a mutual distance of 1.4nm. This densely packed superstructure has been reported for 2H-Pc and M-Pc molecules on various surfaces. Figure 3a shows that molecular islands with this superstructure may coexist with the honeycomb lattice and cover wide areas (Figure 3b). Molecules within this nearly square superstructure exhibit a uniform orientation with one molecular axis aligned with $\langle 11\bar{2}0\rangle$. The presence of the square molecular array implies a superstructure that is incommensurate with the graphene lattice, that is, 2H-Pc molecules occupy all available graphene sites. Therefore, the template effect of the moiré pattern as observed at low coverage is no longer operative. The experimental data do not evidence any influence of the moiré lattice on the densely packed molecular assembly. In particular, large close-packed domains (Figure 3b) appear flat without modulation of the apparent height of individual molecules. A similar structure was reported for Co-Pc on graphene-covered Ir(111) [56], which indicates that the presence or absence of a metallic atom at the Pc center does not modify the molecular superlattice. We suggest that the close-packed arrangement of 2H-Pc at higher coverage results from intermolecular interactions that dominate the molecule–substrate coupling. This suggestion is in accordance with a previous report on 2H-Pc adsorbed on the more reactive graphene-covered Ru(0001) surface [55]. Intermolecular interactions were demonstrated to induce the transition from a kagome lattice to a square lattice upon increasing the coverage.

## 3. Materials and Methods

The experiments were performed using an STM operated in ultrahigh vacuum ($10^{-9}\;\mathrm{Pa}$) and at low temperature (7K). The Ir(111) surface was cleaned by repeated Ar${}^{+}$ bombardment and annealing. Graphene was prepared by thermal decomposition of C${}_{2}$H${}_{4}$ (purity 99.9%) on Ir(111) [64,65,66]. 2H-Pc molecules were sublimated from a heated W crucible and adsorbed on Ir(111) in the submonolayer range at room temperature. All STM images were acquired at constant current with the bias voltage applied to the sample.

## 4. Conclusions

Molecular superstructures of 2H-Pc on graphene-covered Ir(111) unravel the coverage-dependent transition from assemblies that are guided by the graphene moiré pattern to arrangements that are determined by the intermolecular coupling. A honeycomb lattice of 2H-Pc molecules is formed at intermediate coverage, which reflects the template effect of the moiré pattern. The mismatch between the moiré periodicity and the molecular dimensions prevents the formation of a kagome lattice. For a coverage in the vicinity of the closed molecular layer 2H-Pc adopt a close-packed arrangement that is independent of the graphene–metal complex.
